# Hyaluronic Acid Injectable Gel VYC-25L Is Safe and Effective for Adults Seeking Chin Enhancement to Correct Chin Retrusion: Results From a Real-world Evidence Study in China

**DOI:** 10.1007/s00266-024-04572-3

**Published:** 2025-02-26

**Authors:** Yimin Liang, Wang Zhan, Yun Xie, Danru Wang, Qingfeng Li, Grace Zhao, Smita Chawla

**Affiliations:** 1 Shanghai Ninth People’s Hospital, No. 639, Zhizaoju Road, Shanghai, 200011 People’s Republic of China; 2 Hainan General Hospital, Qionghai City, Hainan People’s Republic of China; 3 Allergan Aesthetics, Shanghai, People’s Republic of China; 4 Allergan Aesthetics, Irvine, CA USA

**Keywords:** Asian, Chin, Esthetics, Hyaluronic acid, Injection, Jaw

## Abstract

**Background:**

Chinese individuals may seek chin enhancement to address esthetic perceptions regarding chin retrusion. Because studies of the injectable hyaluronic acid gel VYC-25L (Allergan Aesthetics, an AbbVie Company, Irvine, CA) contained few Asian subjects and none from China, its effects on chin retrusion in Chinese individuals are unclear. This 12-month, real-world evidence study evaluated the safety and effectiveness of VYC-25L for chin enhancement in Chinese adults.

**Methods:**

At Hainan Bo’ao Super Hospital, 2 cohorts of prospectively and retrospectively enrolled adults received VYC-25L treatment (maximum 4 mL). Cohort 1 only underwent 3D imaging before and after treatment for digital analysis. The primary effectiveness measure was mean change from baseline in glabella–subnasale–pogonion (G–Sn–Pog) angle at month 3 based on 3D facial images. Both cohorts completed the Global Aesthetic Improvement Scale (GAIS; subjects and investigators) and a satisfaction questionnaire (subjects). Injection site response (ISR) and adverse events were recorded.

**Results:**

Of 90 subjects enrolled (cohort 1, n=36; cohort 2, n=54), 89 completed the study. Mean change from baseline in G–Sn–Pog angle at month 3 was 3.19 degrees (95% CI, 2.55–3.83; *P* < 0.001 vs 0), with improvement maintained through 12 months. Month 3 “improved”/“much improved” GAIS responder frequencies were 94.3% (investigators) and 97.1% (subjects); 91.5% of subjects were “satisfied”/“very satisfied” with treatment at month 3. Most (>70%) ISRs were mild or moderate in severity. Four treatment-related adverse events occurred (all mild).

**Conclusion:**

VYC-25L was safe and effective for enhancement of the chin and jaw in Chinese adults.

**Level of Evidence III:**

This journal requires that authors assign a level of evidence to each article. For a full description of these Evidence-Based Medicine ratings, please refer to the Table of Contents or the online Instructions to Authors www.springer.com/00266.

## Introduction

Chin shape and projection contribute to the overall esthetic profile of the face, an individual’s psychological well-being, and societal perceptions of attractiveness [1, 2]. Studies have documented that Chinese individuals prefer a narrow, pointy, anteriorly projected chin for women and a round, narrow, but less-pointy chin for men to a facial shape with a square jaw angle and a wide or flat chin [3, 4]. These cultural perceptions of beauty, as well as age-related changes of the lower face such as chin ptosis, may prompt individuals to seek esthetic treatment of the chin and lower jawline [1, 4]. However, filler treatments or comparable treatments for this indication are unavailable in China.

Hyaluronic acid (HA) injectable fillers are nonsurgical esthetic treatment options that are commonly used for rejuvenating areas of the lower face, including the chin and jawline [5–7]. The 25-mg/mL HA injectable gel VYC-25L (Allergan Aesthetics, an AbbVie Company) was designed for creating and restoring facial volume, including chin augmentation and jawline definition, as its higher concentration of HA permits sculpting, contouring, and shaping. VYC-25L is safe and effective for enhancing the chin and jawline, with results lasting through 1 year [2, 8–11].

The clinical trials of VYC-25L conducted in Europe and the USA enrolled a limited number of subjects of Asian descent (0%–12.5% of the study population) [2, 8, 10]. It is important to understand the safety and effectiveness of VYC-25L in different ethnicities; thus, the aim of this real-world experience study was to evaluate the safety and effectiveness of VYC-25L in clinical practice among Chinese adults seeking chin enhancement to correct chin retrusion at Hainan Bo’ao Super Hospital, where VYC-25L has been approved by the Hainan Drug Authority for named patient use.

## Methods

### Study Design

**Fig. 1:**
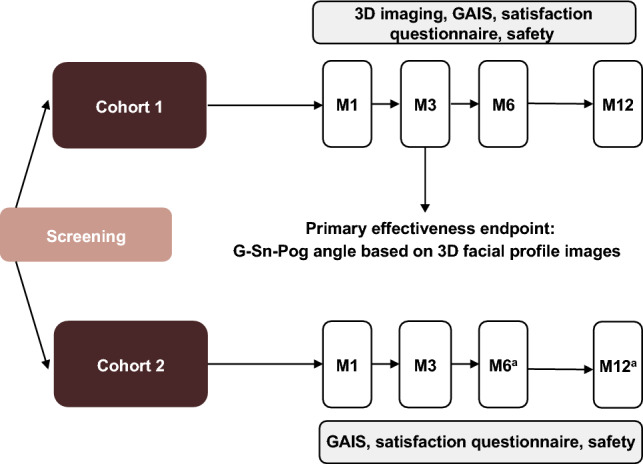
Study design. 3D, three-dimensional; G–Sn–Pog, glabella–subnasale–pogonion; GAIS, Global Aesthetic Improvement Scale; M, Month. ^a^Follow-up conducted via phone; GAIS results not collected

This 12-month, open-label study (NCT04687046) was conducted from February 2021 to February 2022 at Bo’ao Super Hospital in Hainan, China. Two cohorts of subjects were prospectively or retrospectively enrolled and analyzed (Fig. 1). In cohort 1, subjects received VYC-25L treatment and underwent 3D imaging before and after treatment for digital analysis assessments. Follow-up visits were conducted onsite at months 1, 3, 6, and 12 after treatment. In cohort 2, which did not undergo 3D imaging and was included to ensure sufficient data for comprehensive effectiveness and safety assessments, subjects received VYC-25L treatment; follow-up visits were conducted on-site at months 1 and 3 and follow-up phone calls were conducted at months 6 and 12. Comparator treatments were not evaluated in this study, as there were no HA injectable fillers for chin enhancement approved by the Center for Medical Device Evaluation under the National Medical Products Administration of China during the study period.

This study was approved by an independent ethics committee, the Hainan Bo’ao Super Hospital Ethics Committee, and was conducted in compliance with the Declaration of Helsinki and applicable laws and regulations, including the China Medical Device Good Clinical Practice guidelines. All subjects provided written informed consent prior to any data collection.

### Subjects

Eligible subjects were Chinese adults aged 18 years and older who had chin retrusion that could be improved with HA injectable fillers, as assessed by the treating investigator. Eligible subjects were also seeking chin enhancement, and this was based on the subject’s assessment of satisfaction with chin at baseline (no subjects reported being satisfied or very satisfied with their chin). Finally, subjects had received or planned to receive VYC-25L treatment at Hainan Bo’ao Super Hospital. Individuals were excluded from participation if they had received prior chin or jaw surgery, including cartilage grafts or implantation of any biomaterials; permanent/semipermanent fillers or fat injections in the treatment area; or temporary fillers other than VYC-25L injected in the treatment area within 12 months before enrollment. Other exclusion criteria were presence of untreated epilepsy or porphyria; current cutaneous inflammation or infection in the treatment area; were pregnant or breastfeeding; history of hypersensitivity to lidocaine or any amide-based anesthetics, HA, or streptococcal protein; or a tendency to develop hypertrophic scarring.

### Treatment Administration

VYC-25L was injected using a 27-gauge, 0.5-inch needle to the subcutaneous and/or supraperiosteal planes of the chin and prejowl sulci. After application of topical pretreatment anesthesia, subjects received a single VYC-25L treatment of up to 2.0 mL per area, which could include the pogonion, mentum, and right and left prejowl sulci, for a maximum total volume of 4 mL. The volume injected for each subject was determined by the treating physician and was based on clinical experience.

### Effectiveness

**Fig. 2:**
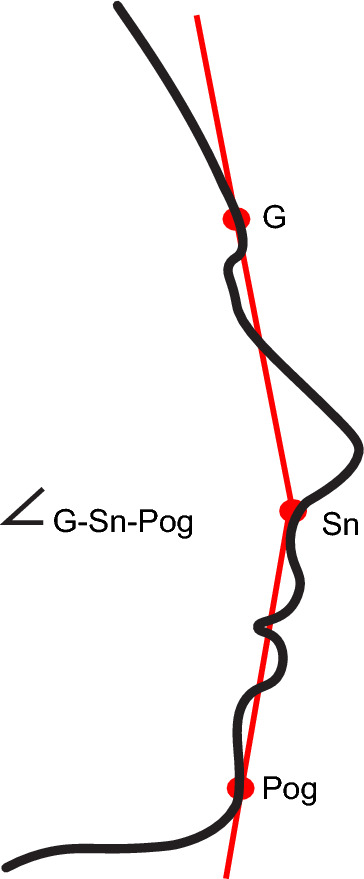
Schematic of the glabella–subnasale–pogonion angle (primary effectiveness endpoint). G, glabella; Pog, pogonion; Sn, subnasale

The primary effectiveness endpoint, measured in cohort 1 alone, was mean change from baseline to month 3 in the glabella–subnasale–pogonion (G–Sn–Pog) angle, as measured by a blinded image analysis technician. At screening, the positions of the glabella, subnasale, and pogonion were set by the blinded technician using 3D facial profile images. At each follow-up timepoint after treatment, the blinded technician aligned the 3 landmarks based on the positions set in the digital images captured at screening to calculate the G–Sn–Pog angle using an algorithm developed by Canfield Scientific (Parsippany, NJ; Fig. 2).

Secondary effectiveness endpoints (pooled cohorts 1 and 2) were responder status at month 3 for the investigator- and subject-assessed Global Aesthetic Improvement Scale (GAIS) ratings of the chin and jaw area. A GAIS responder was defined as a having a rating of “improved” or “much improved” on the 5-point GAIS (2 = much improved, 1 = improved, 0 = no change, −1 = worse, −2 = much worse). Investigators assessed esthetic improvement of the subject’s chin and jaw area by comparing the subject at each follow-up visit with the profile view photographs obtained at baseline. Subjects assessed the level of improvement by comparing their own appearance in a 3-fold mirror at each follow-up visit with the photographs obtained at baseline.

Other effectiveness endpoints were mean changes from baseline in the G–Sn–Pog angle, chin volume, and chin (pogonion) projection as assessed using digital analysis of 3D images at each follow-up visit (cohort 1); investigator and subject GAIS assessments of the chin and jaw area at each follow-up visit (pooled cohorts 1 and 2); and subject assessment of satisfaction with the chin and satisfaction with treatment, each evaluated on a 5-point scale (very dissatisfied, dissatisfied, neutral, satisfied, very satisfied) at each study visit (pooled cohorts 1 and 2).

### Safety

Presence of injection site response (ISR) was recorded daily in a safety diary for 30 days following treatment. The subject’s safety diary listed the following ISRs (with corresponding severity of none, mild, moderate, or severe) previously reported with HA filler injections: redness, pain after injection, tenderness to touch, firmness, swelling, lumps/bumps, bruising, itching, and discoloration (not redness or bruising). Adverse events (AEs) were recorded based on physician observations and inquiries during each scheduled follow-up visit.

### Statistical Methods

Sample size determination for G–Sn–Pog angle data collection (cohort 1) was based on results of a previously published prospective study [2]. Using investigator-assessed GAIS as the anchor measure, a post hoc analysis of data from this previous study indicated that a 1.6-degree change in G–Sn–Pog angle was clinically meaningful. Assuming a mean change of 1.6 degrees with an SD of 2.1, it was determined that 21 subjects were required to detect the change with a power of 90% at the 2-sided 5% significance level based on a paired *t* test. Allowing for potential dropouts, a minimum of 30 subjects was targeted for enrollment into cohort 1. Calculations were performed with nQuery 4.0 (Dotmatics, Boston, MA).

The intention-to-treat population consisted of all subjects who received VYC-25L treatment and had at least 1 post-treatment effectiveness assessment. The safety population consisted of all subjects who received VYC-25L treatment and had at least 1 post-treatment safety assessment. The primary effectiveness endpoint was summarized with descriptive statistics and 95% CIs; mean change in the G–Sn–Pog angle at month 3 was compared with 0 using a 2-sided paired *t* test performed at the 5% significance level. For secondary effectiveness endpoints, responder rates and 95% CIs were provided for investigator and subject GAIS assessments at month 3. Other effectiveness endpoints were summarized with descriptive statistics. The ISRs were summarized with categoric descriptive statistics for severity and continuous descriptive statistics for duration.

## Results

### Subjects

Of 90 subjects enrolled and treated (cohort 1, n=36; cohort 2, n=54), 89 (98.9%) completed the study up to month 12. One subject from cohort 1 who was lost to follow-up was discontinued from the study. Mean age of the intention-to-treat population was 33.2 (SD, 6.8; range, 20–58) years, and most subjects were women (85.6%). All subjects received treatment in the pogonion; most also received treatment in the mentum (94.4%) and right and left prejowl sulci (95.6% each). Most subjects (94.4%) received treatment in both the subcutaneous and supraperiosteal planes. For all treatment areas, the most common injection techniques were bolus (pogonion: 52.2%; mentum: 51.1%; right prejowl sulcus: 50.0%; left prejowl sulcus: 50.0%) and vertical column (pogonion: 46.7%; mentum: 42.2%; right prejowl sulcus: 44.4%; left prejowl sulcus: 44.4%). Median volume injected was 4.0 (range, 2.2–4.0) mL, with median injection volumes of 1 mL in the pogonion, 0.8 mL in the mentum, and 1 mL each in the right and left prejowl sulci.

### Effectiveness

**Fig. 3:**
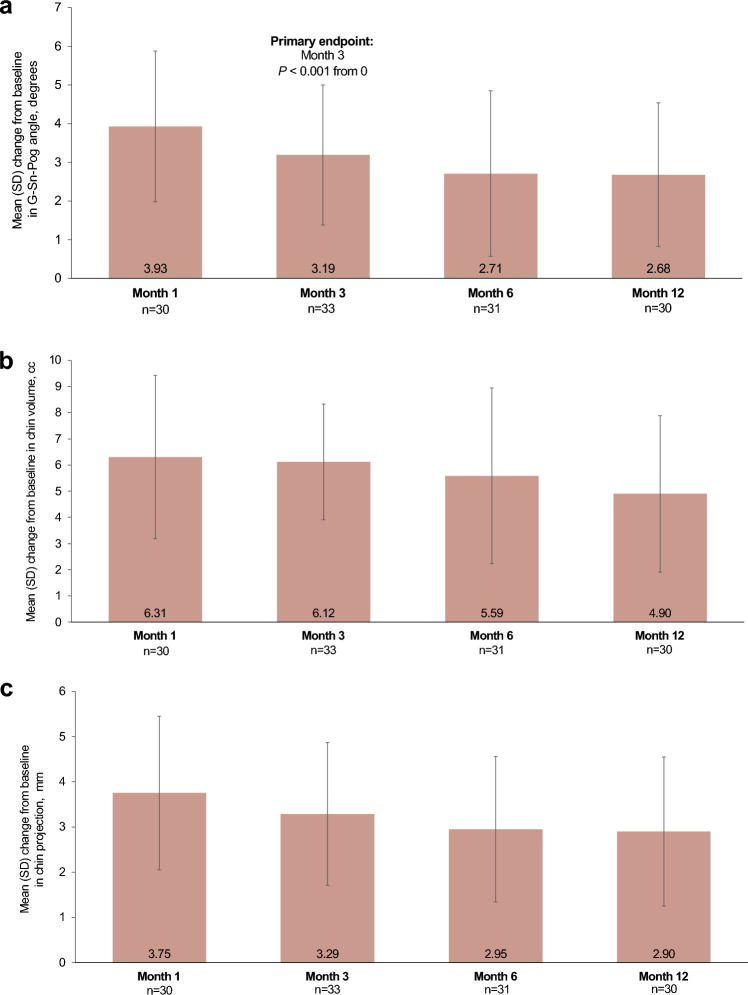
Mean changes from baseline at all timepoints in (**a**) the glabella–subnasale–pogonion angle, (**b**) chin volume, and (**c**) chin (pogonion) projection

Mean G–Sn–Pog angle at month 3 was larger than at baseline (167.81 vs 164.56 degrees, respectively), resulting in a statistically significant mean change from baseline to month 3 of 3.19 degrees (95% CI, 2.55–3.83; *P* < 0.001 vs 0), which met the primary endpoint for effectiveness (Fig. 3a) and indicates a clinically meaningful improvement (≥1.6 degrees). The change in mean G-Sn-Pog angle from baseline to month 3 was normally distributed based on the Shapiro-Wilk normality test (*P* = 0.2586).

**Fig. 4:**
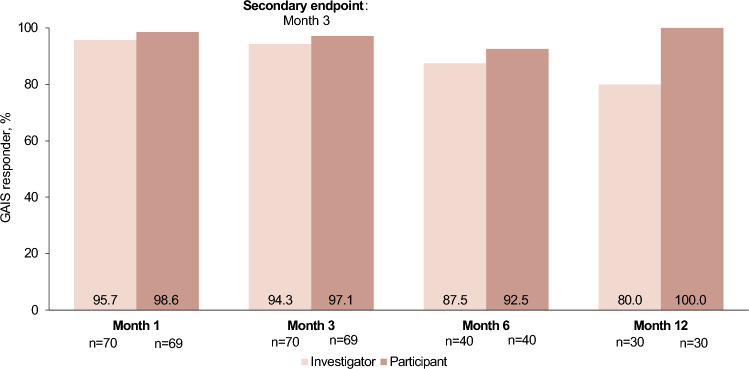
Investigator- and subject-assessed GAIS responder rates of the chin and jaw areas. A GAIS responder is defined as a subject with ratings of “improved” or “much improved” at 3 months. GAIS, Global Aesthetic Improvement Scale

**Fig. 5:**
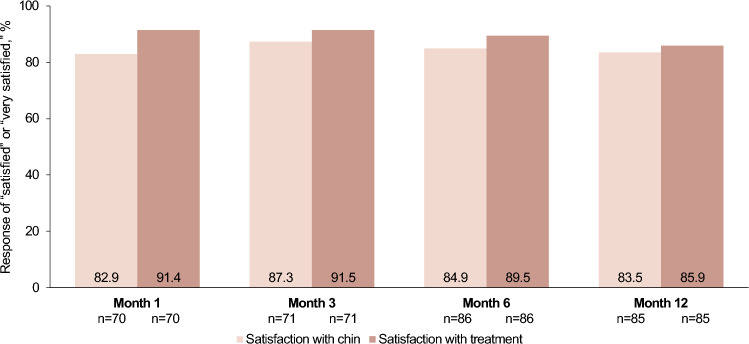
Levels of subject satisfaction with chin area and treatment

Results of the other effectiveness endpoints were also favorable. Investigator and subject GAIS responder rates for the chin and jaw area at month 3 were 94.3% (95% CI, 86.0–98.4) and 97.1% (95% CI, 89.9–99.7), respectively, and remained high throughout the study (Fig. 4). Although none of the subjects reported being “satisfied” or “very satisfied” with their chin at baseline, the majority (>82%) reported being “satisfied” or “very satisfied” with their chin at all post-treatment evaluations (Fig. 5). In addition, the majority of subjects reported being “satisfied” or “very satisfied” with treatment throughout the study (Fig. 5). Mean changes from baseline in G–Sn–Pog angle, chin volume, and chin projection demonstrated substantial clinical improvements at all follow-up visits (Fig. 3b–c).

**Fig. 6:**
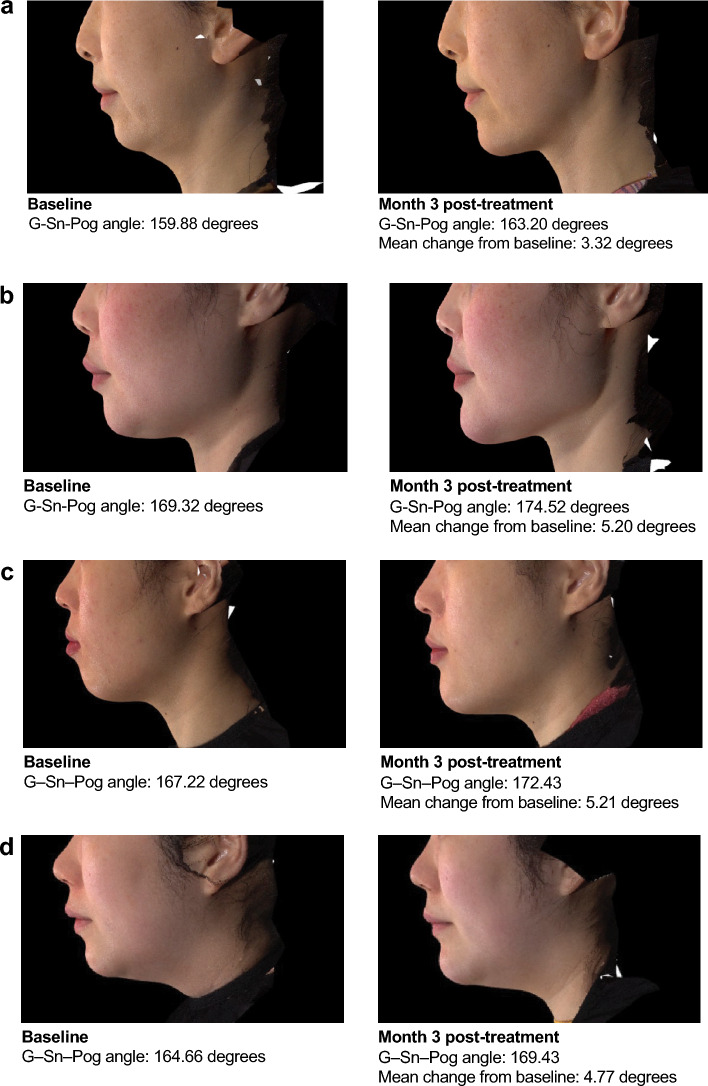
Representative photographs of subjects before and after receiving VYC-25L (Juvéderm Volux XC; Allergan Aesthetics, an AbbVie Company, Irvine, CA) treatment. (**a**) A woman aged 30 years at baseline and 3 months after receiving VYC-25L 4.0 mL. (**b**) A woman aged 39 years at baseline and 3 months after receiving VYC-25L 3.2 mL, (**c**) A woman aged 29 years at baseline and 3 months after receiving VYC-25L 4.0 mL, (**d**) A woman aged 34 years at baseline and 3 months after receiving VYC-25L 4.0 mL. G–Sn–Pog, glabella–subnasale–pogonion

Photographs of 4 representative subjects before and after VYC-25L treatment show the changes in chin projection achieved with an increase in the G–Sn–Pog angle of 3.32 (Fig. 6a), 5.20 (Fig. 6b), 5.21 (Fig. 6c), and 4.77 degrees (Fig. 6d).

### Safety

**Fig. 7:**
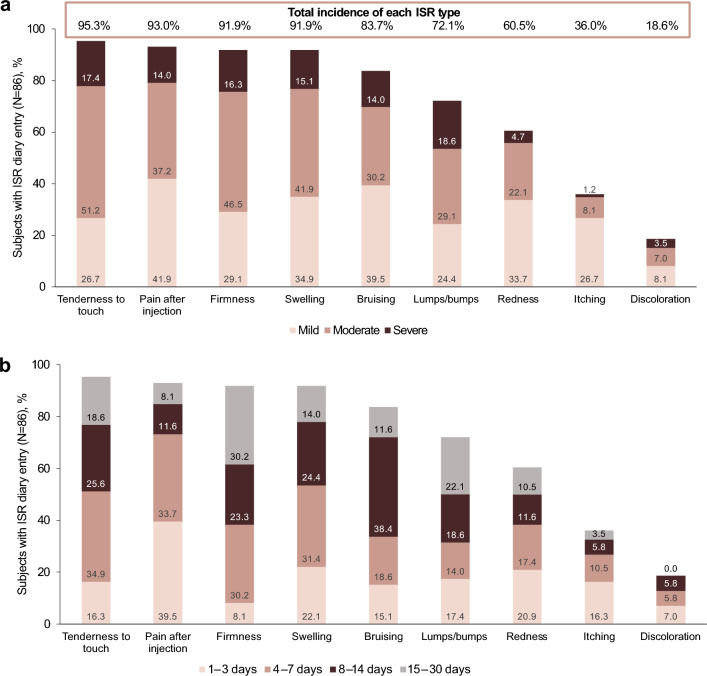
Presence of ISRs ≤30 d of treatment by (**a**) incidence and severity and (**b**) duration. ISR, injection site response

No device/needle problems or malfunctions were reported during the study. All 86 subjects who completed their 30-day diaries reported at least 1 ISR, with the most common being tenderness to touch (95.3%), pain after injection (93.0%), firmness (91.9%), and swelling (91.9%; Fig. 7a). Overall, ISR severity was mild for 20.9% of subjects, moderate for 50.0%, and severe for 29.1%. The majority (57.0%) of ISRs resolved within 2 weeks (Fig. 7b). Sixteen (18.6%) subjects reported ISRs that were ongoing 30 days after treatment, most commonly lumps/bumps (n=11), firmness (n=7), and tenderness to touch (n=4). Ongoing ISRs had a median duration of 63.5 (range, 34–366) days.

During follow-up visits, physicians observed that of 90 subjects, 8 (8.9%) experienced 12 treatment-emergent AEs (TEAEs). Three (3.3%) subjects experienced 4 treatment-related TEAEs. All 4 treatment-related TEAEs occurred at the injection site (swelling [n=3 (3.3%)] and pain [n=1 (1.1%)]), were mild in severity, and spontaneously resolved without sequelae in less than 1 week. None led to study discontinuation or death. There were no treatment-related serious AEs; 3 serious AEs not related to treatment and requiring hospitalization were experienced by 3 subjects (rotator cuff syndrome, thyroid cancer, schwannoma).

## Discussion

In 2021, use of HA soft tissue fillers was the second most commonly performed nonsurgical esthetic procedure worldwide, with approximately 5.3 million procedures performed, representing an increase of more than 30% from the previous year [12]. Although use of HA fillers for lower face contouring has been described in the literature [13, 14], few studies focusing on subjects of Asian descent have investigated the safety and effectiveness of HA fillers for treating chin retrusion [7]. In addition, VYC-25L treatment patterns in the clinical setting of chin retrusion have not been previously evaluated in real-world evidence studies.

The results of the present real-world evidence study demonstrate the safety and effectiveness of VYC-25L treatment for Chinese adults seeking chin enhancement to correct chin retrusion in clinical practice. The primary and secondary endpoints were met. Specifically, at 3 months post-treatment, mean change from baseline in the G–Sn–Pog angle was significantly improved from 0, indicating an increase in the volume and projection of the chin, and the investigator- and subject-assessed GAIS responder rates were high. Improvements in the G–Sn–Pog angle and GAIS were maintained up to 12 months post-treatment and were complemented by high rates of subject satisfaction with their chin and their treatment throughout the study. Treatment with VYC-25L posed few safety issues because ISRs were mostly mild and moderate in severity, and the majority of the reported ISRs resolved within 2 weeks. Three subjects experienced treatment-related TEAEs, all of which were mild in severity, occurred at the injection site, and resolved without sequelae in less than 1 week.

The strength of this study was its combination of objective 3D imaging measurements and patient-reported outcomes in a real-world clinical setting. The G–Sn–Pog angle is clinically useful for the objective evaluation of changes in the projection of the chin based on digital image analysis [8], potentially eliminating bias from assessment of the primary endpoint. Although the G–Sn–Pog angle and rates of patient satisfaction with treatment gradually declined over time, subject-assessed GAIS ratings remained high throughout the study (eg, 100% at month 12), suggesting that durable long-term improvement was perceived by the subjects.

The results of the present study align with those of the pivotal VYC-25L studies in Europe and the USA. In the European prospective, randomized, controlled, 18-month pivotal study assessing VYC-25L treatment for restoring and creating volume in the chin and jawline in individuals with chin retrusion, a similar volume of VYC-25L was used to treat several areas of the chin [2, 8]. Mean change from baseline in the G–Sn–Pog angle was significantly greater in subjects treated with VYC-25L compared with no-treatment controls at months 1 (2.73 vs −0.21 degrees, respectively) and 3 (2.12 vs −0.38 degrees, respectively; *P* < 0.0001) [2], exceeding the 1.6-degree threshold for clinically meaningful improvement and reflected in high investigator and subject GAIS responder rates and patient satisfaction rates. A similar safety profile to that in the present study was observed in the European pivotal study [2]. The US-based randomized, controlled, evaluator-blinded 12-month study assessed VYC-25L treatment for restoring jawline definition; chin injections were permitted to provide a balanced overall esthetic result [10, 11]. Approximately 70% of subjects received VYC-25L injections in the chin. Similar to the findings of the present study, investigator and subject GAIS responder rates, as well as patient satisfaction rates, were high up to month 12 in the US study [10, 11].

The present study had several limitations. The subjects were mostly women, and their mean age of 33 years was younger than the mean ages of 46 and 59 years in the pivotal studies conducted in Europe and the USA, respectively [2, 10], thereby limiting generalizability of the results across sex and age. To capture current clinical practice data, an open-label, noncomparative study design was used; however, this study design may have introduced subjectivity and biases, especially on the subjective rating scales. The results of this noncomparative study are descriptive and should be considered as hypothesis-generating. The study was also limited to a single site/institution in Hainan Province in China. However, multiple investigators at the institution provided treatment, and some subjects traveled from outside Hainan Province to receive treatment.

## Conclusion

Treatment with VYC-25L was safe and effective in the enhancement of the chin and jaw area in Chinese adults with chin retrusion. Improvements in chin projection and jawline, as measured by objective 3D imaging and calculations of the G–Sn–Pog angle and by subjective patient-reported outcomes, were observed at least to 1 year.

## Data Availability

AbbVie is committed to responsible data sharing regarding the clinical trials we sponsor. This includes access to anonymized, individual, and trial-level data (analysis data sets), as well as other information (e.g., protocols, clinical study reports, or analysis plans), as long as the trials are not part of an ongoing or planned regulatory submission. This includes requests for clinical trial data for unlicensed products and indications. This clinical trial data can be requested by any qualified researchers who engage in rigorous, independent scientific research, and will be provided following review and approval of a research proposal and Statistical Analysis Plan (SAP) and execution of a Data Sharing Agreement (DSA). Data requests can be submitted at any time after approval in the US and Europe and after acceptance of this manuscript for publication. The data will be accessible for 12 months, with possible extensions considered. For more information on the process, or to submit a request, visit the following link: https://vivli.org/ourmember/abbvie/ then select “Home”.
