# Correction: Hyaluronic Acid Injectable Gel VYC-25L Is Safe and Effective for Adults Seeking Chin Enhancement to Correct Chin Retrusion: Results From a Real-world Evidence Study in China

**DOI:** 10.1007/s00266-025-05001-9

**Published:** 2025-06-05

**Authors:** Yimin Liang, Wang Zhan, Yun Xie, Danru Wang, Qingfeng Li, Grace Zhao, Smita Chawla

**Affiliations:** 1 Shanghai Ninth People’s Hospital, No. 639, Zhizaoju Road, Shanghai, 200011 People’s Republic of China; 2 Hainan General Hospital, Qionghai City, Hainan People’s Republic of China; 3 Allergan Aesthetics, Shanghai, People’s Republic of China; 4 Allergan Aesthetics, Irvine, CA USA

**Correction to:** Aesth Plast Surg 10.1007/s00266-024-04572-3

The article “Hyaluronic Acid Injectable Gel VYC-25L Is Safe and Effective for Adults Seeking Chin Enhancement to Correct Chin Retrusion: Results From a Real-world Evidence Study in China,” written by Liang et al, was originally published under exclusive license to Springer Science+Business Media, LLC, part of Springer Nature and International Society of Aesthetic Plastic Surgery. As a result of the subsequent decision to publish the article under the open access model, the article’s copyright notice was changed on 19 May 2025 to © The Author(s) 2025 and the article is now distributed under a Creative Commons Attribution CC BY: This licence permits use, duplication, adaptation, distribution and reproduction in any medium or format, as long as appropriate credit is given to the original author(s) and the source, a link is provided to the Creative Commons licence, and any changes made are indicated. Please read the full licence for further details at—http://creativecommons.org/licenses/by/4.0/.

